# Members of the *Fusarium fujikuroi* Species Complex Isolated from Asymptomatic Wetland Grasses in Argentina Include Previously Described Species Pathogenic on Cereal Crops and a Novel Species

**DOI:** 10.3390/jof12060444

**Published:** 2026-06-17

**Authors:** Eugenia Cendoya, Cindy J. Romero Donato, María J. Nichea, Sofía A. Palacios, Mark Busman, Robert H. Proctor, María L. Ramirez

**Affiliations:** 1 Instituto de Investigaciones en Micología y Micotoxicología (IMICO), CONICET-Universidad Nacional de Rio Cuarto, Ruta 36 Km 601, Río Cuarto 5800, Córdoba, Argentina; cindyjrd@hotmail.com (C.J.R.D.); juli_nichea@yahoo.com.ar (M.J.N.); spalacios@exa.unrc.edu.ar (S.A.P.); mramirez@exa.unrc.edu.ar (M.L.R.); 2 USDA, Agricultural Research Service, National Center for Agricultural Utilization Research, (UNIT), 1815 N University St., Peoria, IL 61604, USA; mark.busman@usda.gov (M.B.); robert.proctor@usda.gov (R.H.P.)

**Keywords:** Poaceae, *Fusarium fujikuroi* species complex, phytopathogens, new taxon

## Abstract

The floodplains of the Paraná and Paraguay rivers form the Chaco wetland, one of the most species-rich plant ecosystems in Argentina. Because wild grasses can serve as reservoirs of fungal species that cause disease and mycotoxin contamination of cereal crops, we examined asymptomatic, wild grasses from the Chaco wetlands for the presence of the genus *Fusarium*, which includes multiple species that cause agriculturally important diseases and/or mycotoxin contamination of crops. We focused our efforts on the identification and characterization of the multispecies lineage known as the *Fusarium fujikuroi* species complex (FFSC). Using morphological traits and partial DNA sequences of the *TEF1* gene, we determined that 58 isolates recovered from the grasses were members of FFSC. Fifty of the isolates were identified as one of six FFSC species, including the economically important plant pathogenic species *F. proliferatum*, *F. subglutinans*, and *F. verticillioides*. To our knowledge, two of the species, *F. anthophilum* and *F. pseudocircinatum*, have not been reported previously in Argentina. Our analyses also indicated that eight of the FFSC isolates were a novel species, herein described as *Fusarium varsavskyanum*. A polymerase chain reaction (PCR) assay and genome sequence data indicate that each isolate of *F. varsavskyanum* isolate had only one mating type idiomorph (MAT1-1 or MAT1-2), which suggests that the fungus is heterothallic. Genome sequence analysis indicated that *F. varsavskyanum* has the genetic potential to produce, (i) the emerging mycotoxins fusaric acid and beauvericin (or enniatins); (ii) the pigments bikaverin, carotenoids, and fusarubin; and (iii) the plant hormones auxins, cytokinins, and gibberellins. Thus, asymptomatic grasses from the Chaco wetland can harbor *Fusarium* species that in some agroecosystems can cause economically important diseases and/or mycotoxin contamination of crops. It remains to be determined whether the genotypes of *Fusarium* species that occur on the wetland grasses, including *F. varsavskyanum* genotypes, can negatively impact agriculture.

## 1. Introduction

The grass family, Poaceae, is one of the most species-rich families within the kingdom Plantae. Poaceae species are both ecologically and economically important because they are a dominant component of multiple ecosystems, and multiple species are used as cereal crops and in pastures [1]. Approximately 70% of the land area of Argentina is natural grassland [2]. This includes grasslands in the Chaco Wetlands located in the east of Chaco Province. The wetlands are formed by the Paraná and Paraguay river floodplain and are one of the three most biodiverse ecosystems in Argentina. The wetland landscape is a complex of open water, aquatic vegetation, gallery forests as well as grasslands. A diversity of grasses palatable to livestock and a temperate climate make the grassland of the Chaco Wetlands suitable for grazing cattle year-round [3]. Among the most common grass species in the Chaco Wetlands are *Leersia hexandra*, *Luziola peruviana*, *Sorghastrum setosum*, *Spartina argentinensis* and *Cynodon dactylon*. In a previous study, examination of 175 asymptomatic grass plants representing 12 genera revealed that all the plants were contaminated with *Fusarium* mycotoxins. The predominant mycotoxins were zearalenone (ZEA), T-2 toxin, and HT-2 toxin. Other mycotoxins/metabolites that occurred less frequently and/or at lower levels were beauvericin, fumonisin B_1_, and equisetin [4]. All the metabolites noted above are reported to be produced by one or more species of *Fusarium* [5,6].

A mycological analysis of the Chaco Wetland grasses revealed that *Fusarium* was recovered from 60 to 100% of plants per sample site [4]. Analysis of the *Fusarium* isolates recovered from asymptomatic grasses revealed that some were T-2 and HT-2 toxin-producing species, including *F. chaquense*, a recently described member of the multispecies lineage known as the *Fusarium sambucinum* species complex [7]. Another species recovered was a member of the *F. incarnatum*-*equiseti* species complex and was likely responsible for ZEA contamination observed in the wetland grasses [8]. Morphology-based analyses revealed that other isolates recovered from the Chaco Wetland grasses were members of the *F. fujikuroi* species complex (FFSC).

FFSC is one of the most species-rich lineages within the genus *Fusarium*. Comparative morphological and molecular phylogenetic studies indicate that the FFSC comprises over 60 phylogenetically distinct species that can be further resolved into three clades—the African, American and Asian clades—which were proposed to have diverged from one another on different continents after fragmentation of the supercontinent Gondwana [9,10,11,12,13,14,15]. FFSC is a global threat to food/feed security and safety because many of its species cause economically important crop diseases and/or produce mycotoxins that are health hazards to humans and livestock [6,14,16,17]. Crop diseases caused by fungi in the FFSC include ear rot, stalk rot and seedling blight of maize, pitch canker of pine trees, bakanae disease of rice, pokkah boeng disease of sugarcane, and mango malformation [17].

Multiple members of the FFSC can occur as both pathogens and endophytes of plants from natural and agricultural environments [18]. For example, *F. fujikuroi* causes bakanae of rice but it also forms endophytic associations with aquatic plants of the genus *Echinochloa* [19]; *F. circinatum* causes pitch canker of pine trees but it is also an endophyte of wild grasses [20,21]; and *F. giganteum* is a pathogen of maize but also an endophyte of the forage grass *Panicum maximum* [22]. There are also FFSC members that are endophytes of grasses in natural environments but not reported to be plant pathogens. These FFSC members include the *Andropogon* and *Sorghastrum* endophyte *F. konzum* [23] the *Coix gasteenii* endophyte *F. coicis*, the *Sorghum interjectum* endophyte *F. tjaebata* [24] and the *Brachiaria* spp. endophytes *F. caapi* and *F. brachiariae* [25]. The fact that some crop pathogens are endophytes in other plants has important agricultural implications. For example, endophyte-infected asymptomatic plants are potential reservoirs of inoculum that could initiate disease epidemics in crops and/or potential sources of genetic diversity of plant pathogens [26].

The objective of the current study was to determine species diversity among 58 *Fusarium* isolates previously recovered from asymptomatic wild grasses from the Chaco Wetlands. In the previous study, the isolates were identified by morphological characters as members of the FFSC. In the current study, we used DNA-based analyses to determine the species identities of the fungal isolates and to formally describe and characterize the biology and chemistry of a novel species discovered among the isolates.

## 2. Materials and Methods

### 2.1. Fusarium Isolates

The *Fusarium* isolates (*n* = 58) analyzed in the current study were previously isolated from the aerial parts asymptomatic grasses (Poaceae). The grasses were collected in July 2011 and September and February 2014 from the Chaco Wetlands (Chaco Province), Argentina. All the isolates were morphologically characterized according to Leslie and Summerell [27] as members of the FFSC. The strains are deposited at the Research Institute on Mycology and Mycotoxicology (IMICO, UNRC-CONICET), Universidad Nacional de Rio Cuarto culture collection (RC). Cultures are maintained in 15% glycerol at −80 °C. Two isolates (RC-J82 and RC-J132 = type) of the novel species described herein were also deposited at the United States Department of Agriculture’s ARS Culture Collection (NRRL) as accessions NRRL 64882 and NRRL 64883 (Table 1).

### 2.2. Phylogenetic Analysis and Whole-Genome Sequencing

Isolates were grown in complete medium [27] and incubated on an orbital shaker (150 rpm, New Brunswick Scientific CO., INC, Edison, NJ, USA) for at least three days at 25 ± 1 °C. The resulting mycelia were harvested by filtration through non-gauze milk filters (Ken AG, Ashland, OH, USA). Excess water was removed by blotting mycelia between clean paper towels, and dried mycelia were stored frozen at −20 °C. DNA extraction was performed with the acetyl-trimethyl ammonium bromide method [26]. DNA concentration was estimated using a NanoDrop 2000 instrument (Thermo Fisher Scientific Inc., Waltham, MA, USA).

Partial sequence of the translation elongation factor-1α gene (*TEF1*) was analyzed following PCR amplification with the primers and conditions described by O’Donnell et al. [10]. All PCR products generated were separated by electrophoresis through 1.5% (*w*/*w*) agarose gels stained with ethidium bromide (5 μg/mL), visualized and photographed using a MiniBIS Pro gel documentation system (DNR Bio-Imaging Systems, Jerusalem, Israel) to confirm that a ~700 bp fragment was amplified. Fragments purification and sequencing of both strands was performed commercially (Macrogen, Inc., Seoul, South Korea) using the same primers used for the PCR amplifications. As both DNA strands were sequenced, they were aligned with ClustalW [28], as implemented in the program BioEdit version 7.0.9.0 [29] in order to detect and correct sequencing errors. The *TEF1* sequences of selected strains from each species were deposited in GenBank under the accession numbers indicated in Table 1. Nucleotide sequence comparisons were performed using the Basic Local Alignment Search Tool (BLAST, https://blast.ncbi.nlm.nih.gov, accessed on 30 July 2025) at the National Centre for Biotechnology Information (NCBI). Multiple sequence alignment of the *TEF1* gene was performed using the Web-based program MAFFT (http://mafft.cbrc.jp/aligment/software, accessed on 5 August 2025.) [30]. *TEF1* sequences from reference FFSC strains and other *Fusarium* species obtained from GenBank were included in the analysis (Table 2). Based on this alignment, phylogenetic analyses were performed on selected strains by the maximum parsimony (MP) method using TNT 1.1 (Tree Analysis Using New Technology) [31], maximum likelihood (ML) using PhyML 3.1 [32] and Bayesian inference (BI) using MrBayes 3.2.6 [33]. The MP analysis used the heuristic search option with 1000 random addition sequences with tree bisection reconnection (TBR) branch swapping, saving 10 trees per replicate. Clade stability was assessed by 1000 bootstrap replications. Gaps were treated as “fifth state”. For ML and BI analyses, the best substitution model for the *TEF1* alignment (i.e., TrN + G model) was determined using the Akaike information criterion (AIC) as implemented in jModelTest 2.1.10 [34]. For ML analysis, the robustness of the best tree was evaluated by 1000 bootstrap replications. For BI analysis, two runs with four chains each were run for 10 million generations with a sampling frequency of every 100 generations and the first 25% of trees from each run were discarded as burn-in. *Fusarium oxysporum* NRRL 22902 (AF160312) was used as the outgroup.

For those isolates that could not be identified to species using *TEF1* sequence, partial sequences of the second largest subunit of RNA polymerase (*RPB2*), beta-tubulin (*TUB2*), and calmodulin (*CDM1*) genes were amplified using previously described primers and conditions [35,36,37,38,39] and PCR products were evaluated as described above for *TEF1* amplicons. Phylogenetic analyses of each gene partition and the combined dataset were performed by BI as described above. The TIM3ef + G model was used for *RPB2*, TIM1 + G for *TUB2*, and TrNef + I for *CDM*. For the combined dataset, each gene was treated as a separate partition with independent parameter estimations. The resulting partial sequences of *RPB2*, *TUB2* and *CDM* were deposited in GenBank under accession numbers indicated in Table S1.

Whole genome sequence data of strains NRRL 64882 and NRRL 64883 were generated using a MiSeq platform (Illumina, San Diego, CA, USA) at USDA ARS NCAUR. Strains were grown in liquid GYP medium (2% glucose, 1% peptone, and 0.3% yeast extract) for two days, and the resulting mycelia were harvested by filtration, lyophilized, and ground to a powder. Genomic DNA was extracted from ground mycelia using the Genomic-Tip 20/G protocol (Qiagen, Aarhus, Denmark) and then used to prepare sequencing libraries with the Nextera XT DNA Library Preparation Kit (New England BioLabs, Ipswich, MA, USA). Using CLC Genomics Workbench (CLC Bio-Qiagen, Aarhus, Denmark), MiSeq-generated sequence reads were screened against genome sequences of 84 bacterial species to remove potential contaminating sequence reads, trimmed to remove low-quality data at the ends of reads, and then subjected to de novo assembly using the following parameter settings: word size = 20; bubble size = 50; minimum contig length = 500; auto-detect paired distances = checked; and perform scaffolding = checked. The resulting assemblies for strains NRRL 64882 and NRRL 64883 have been deposited at DDBJ/ENA/GenBank as accessions JBNHW000000000 and JBNHUV000000000, respectively.

To determine which *Fusarium* secondary metabolite biosynthetic genes are present in the genome sequences of strains NRRL 64882 and NRRL 64883, we downloaded nucleotide sequences of genes required for synthesis of 31 *Fusarium* mycotoxins, pigments, plant hormones and other secondary metabolites from the GenBank/National Center for Biotechnology Information (NCBI) or Joint Genome Institute MycoCosm databases. For a given gene cluster, we generally selected sequences from a species in which the cluster is well characterized. The sequences of the genes and species from which they were retrieved are shown in Supplementary File S1. The gene sequences were then used as queries in BLASTn analysis against the assembled NRRL 64882 and NRRL 64883 genome sequences in an in-house database maintained in CLC Genomics Workbench 12.0 (CLC Bio–Qiagen, Aarhus, Denmark), as previously described [40,41].

Full-length sequences of 20 housekeeping genes from the genome sequences of NRRL 64882, NRRL 64883, and strains of 21 other *Fusarium* species in the FFSC were also subjected to phylogenetic analysis (Table S2). The 20 housekeeping genes were selected based on their use in previous investigations of phylogenetic relationships of *Fusarium* species [42,43,44,45]. The 20 genes were retrieved using the BLASTn function [46] in the CLC Genomics Workbench against an inhouse database of genome sequences retrieved from GenBank. Sequences for individual genes, with intron sequences, were aligned using the MUSCLE option in the program MEGA 7.0 [47]. Each alignment was subjected to maximum likelihood analysis using the program IQ-TREE version 1.6.12 with the ultrafast bootstrapping method [48,49]. Alignments for individual genes were concatenated using SequenceMatrix [50], and the resulting concatenated alignment was subjected to partitioned maximum likelihood analysis in IQ-TREE with ultrafast bootstrapping and to gene concordance factor analysis (Supplementary File S2).

### 2.3. Chemical Analysis

To assess the potential of *F. varsavskyanum* to produce mycotoxins and other secondary metabolite, strains NRRL 64882 and NRRL 64883 were grown on V8 Juice agar (20% V8 juice, 0.3% CaCO_3_, 2% agar) at 28 °C. After 7 days, two 5 mm plugs of the resulting cultures were placed in 6-dram vials containing autoclaved maize kernel medium (2.5 g cracked maize kernels and 1.2 mL distilled water). After incubation in the dark for 7 days at 25 °C, the kernel cultures were extracted with 10 mL 86:14 acetonitrile/water for 30 min with shaking. HPLC-MS analysis was performed using a Dionex UltiMate U3000 liquid chromatography system coupled to a QExactive high resolution mass spectrometer equipped with an electrospray ionization (ESI) source (ThermoFisher Scientific, Waltham, MA, USA). Metabolites were separated using a Phenomenex Kinetex 2 mm × 50 mm XB-C18 100A column (2.6 μm particle size, 100 Å pore size, Phenomenex, Torrance, CA)). For analysis of enniatins, beauvericin, bikaverin, bostrycoidin, and fusarubin, elution of metabolites was accomplished in a binary gradient flow of mobile phase A [water/acetic acid (99.7: 0.3 *v*/*v*)] and mobile phase B [methanol/acetic acid (99.7: 0.3 *v*/*v*)], in which the injection volume was 10 μL. The gradient of 20–95% mobile phase B over 5 min was delivered at a flow rate of 0.6 mL/min. The HPLC flow was coupled to the mass spectrometer operated in positive mode utilizing the following parameters: 320 °C capillary temperature, 310 °C heater temperature, and spray voltage of 4.00 kV for positive ESI. For analysis of fusaric acid and moniliformin, the HPLC utilized a gradient of mobile phase A [water/formic acid (99.1: 0.1 *v*/*v*)] and mobile phase B [methanol/formic acid (99.1: 0.1 *v*/*v*)]; injection volume was 10 μL, and the HPLC column was a Waters XBridge 4.6 mm × 150 mm BEH-C18 column (5 μm particle size, 130 nm pore size, Waters Corporation, Milford, MA, USA)). The gradient of 5–95% mobile phase B over 5 min was delivered at a flow rate of 0.8 mL/min. The HPLC flow was coupled to the mass spectrometer operated in negative mode utilizing the following parameters: 320 °C capillary temperature, 310 °C heater temperature, and spray voltage of −4.00 kV for negative ESI. For both positive and negative mode experiments, the mass spectrometer was operated in full MS mode (*m*/*z* range 150/2000 and 70,000 resolution). Quantifications and identifications of each metabolite were performed by comparison to purified standards. Instrument operation and data processing were done using Xcalibur data acquisition and interpretation software (ThermoFisher Scientific, Waltham, MA, USA). Limits of quantitation for fusaric acid, beauvericin, bikaverin, bostrycoidin, fusarubin, moniliformin as well as enniatins A, A1, B, and B1 were 1 ng/μL.

### 2.4. Morphological Characterization of the Novel Species

Morphology of *Fusarium* isolates were examined after 15 days’ growth of cultures initiated from a single conidium. Cultures were grown on carnation leaf agar (CLA) medium at 25 °C under a 12 h light/12 h dark photoperiod with cold white and black, fluorescent lamps. Morphological characters examined included the shape and size of macroconidia produced in sporodochia, the shape and mode of formation of microconidia, including the type of conidiogenous cells, and production of chlamydospores. Measurements and photomicrographs were recorded from a minimum of 20 elements for each structure, using sterile water as mounting medium and a Motic^®^ Panthera L Life Sciences microscope with a built-in Smart CAM digital head and ImageOnDevice System and Images Plus 3.0 software (Motic Electric Group, Xiamen, China). Dimensions are given as the range of measurements with extremes in parentheses followed by mean ± standard deviation (SD). Pigmentation of colonies was determined by growing isolates on potato dextrose agar (PDA) and Spezieller Nährstoffarmer agar (SNA). Color of pigmentation was determined using a standardized color atlas [51].

Mean growth rates at 5, 15, 20, 25, 30 and 35 °C were obtained from diameters of colonies initiated from single spore on PDA (90 mm Petri dishes with 20 mL agar medium) measured after 72 h of incubation in the dark. Three replicate plates for each isolate were used at each temperature [27].

### 2.5. Mating Type Idiomorph and Sexual Crosses

Mating-type idiomorphs (MAT-1 and MAT-2) of eight isolates of the novel species were determined using the PCR protocol described by Montoya-Martínez et al. [52]. Genome sequence data were used to confirm the mating types of two strains, NRRL 64882 and NRRL 64883. Crosses of isolates with opposite mating types were done as described by Klittich and Leslie [53]. Female parents were incubated on carrot agar, while male parents were grown on SNA slants. Petri plates with crosses were incubated at 20 °C under a 12 h white light photoperiod and evaluated weekly over five weeks for the production of perithecia and ascospore exudation. Each isolate was tested as a female and as a male parent in separate heterothallic crosses. All crosses were repeated at least twice. Female-fertile testers E-3693 (MAT-E1) and E-3696 (MAT-E2) of *F. subglutinans* were also used. Tester strains were provided by the Department of Plant Pathology, Kansas State University, Manhattan, KS, USA.

## 3. Results

### 3.1. Determination of Species Identity

Fifty-eight *Fusarium* isolates recovered from asymptomatic grasses growing in the Chaco Wetlands were identified as members of the FFSC based on the following morphological characters: (i) isolates produced both microconidia and macroconidia; (ii) microconidia were borne in chains or false heads and were oval or obovoid; (iii) macroconidia were straight or slightly curved; (iv) chlamydospores were not produced; and (v) colony pigmentation. We did an initial determination of species identity of the isolates using sequences of partial fragments of *TEF1*. The *TEF1* sequence from each isolate was used as a query in BLASTn analysis against the GenBank database with only known species of FFSC selected as references. This analysis provided strong evidence for the species identity of 50 isolates based on high levels (99–100%) of sequence identity. That is, the sequences from 2, 4, 6 and 33 isolates were 100% identical to the FFSC species *F. anthophilum*, *F. subglutinans*, *F. verticillioides*, and *F. proliferatum*, respectively. The sequences from 1 and 4 other isolates were 99 and 99–100% identical to the FFSC species *F. temperatum* and *F. pseudocircinatum*, respectively.

The BLASTn results did not provide clear evidence for the species identity of the eight remaining isolates identified by morphology as members of the FFSC. In the BLASTn analysis, the highest level of identity (97%) of the *TEF1* sequences of the eight isolates was to reference sequences for *F. anthophilum*, *F. bactridioides*, *F. bulbicola*, *F. circinatum*, *F. guttiforme*, and *F. subglutinans*, all of which were members of the American clade of FFSC. The *TEF1* sequences of the eight isolates were 99–100% identical to one another. Furthermore, in the morphological analysis described above, the morphology of the eight isolates was the same. Thus, the results of both the BLASTn and morphological analyses provide evidence that these isolates were all members of a novel species in the American clade of FFSC.

**Figure 1:**
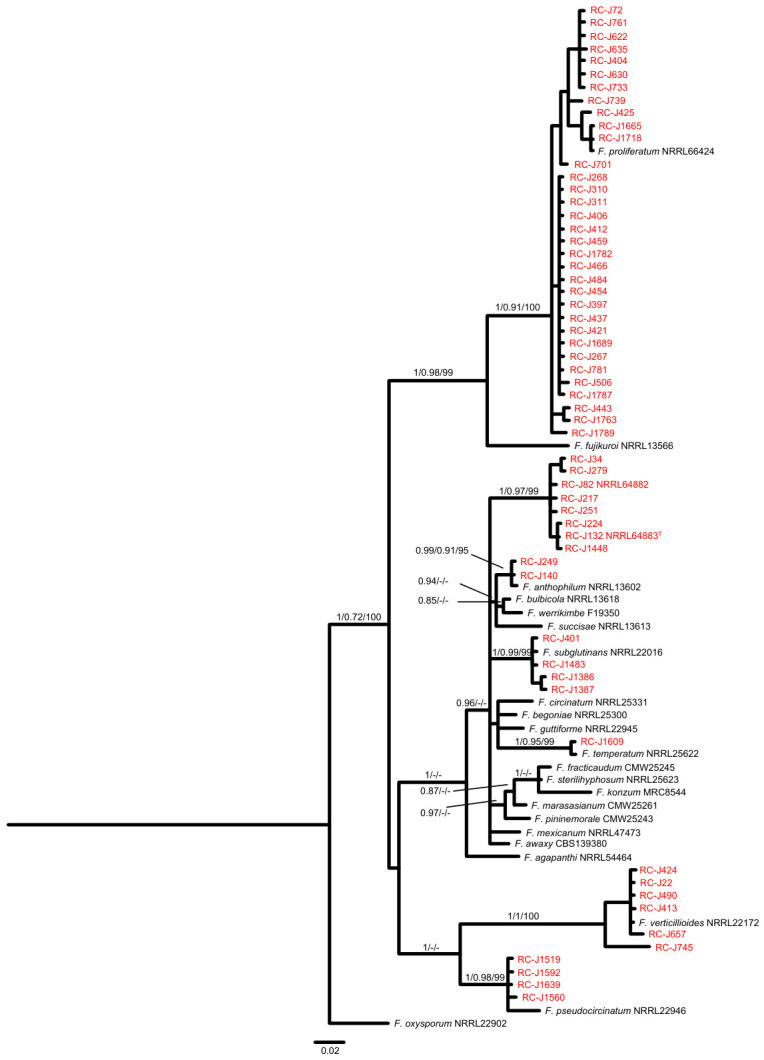
Bayesian inference phylogenetic tree inferred from partial *TEF1* sequences showing the phylogenetic relatedness of *Fusarium* species associated with Poaceae plants with other species of the *Fusarium fujikuroi* species complex. Bayesian posterior probability scores ≥  0.7, followed by ML bootstrap values ≥  0.70, and maximum parsimony bootstrap support values > 70%, are shown at the internodes. The studied strains are in red. *F. oxysporum* NRRL 22902 was used as the outgroup.

To further assess species identities, the *TEF1* sequences of the grass isolates were aligned to FFSC reference sequences retrieved from the Fusarium ID 3.0 and GenBank databases (Table 2), and the resulting alignment was subjected to MP, ML and BI phylogenetic-tree-building analyses. The resulting trees confirmed the species identities determined by BLASTn analysis. That is, the 50 grass isolates identified to species in BLASTn analysis were resolved into exclusive and well supported clades with sequences from references strains of *F. proliferatum*, *F. verticillioides*, *F. subglutinans*, *F. pseudocircinatum*, *F. anthophilum* or *F. temperatum* (Figure 1). The trees also included data for the eight isolates for which the species identity was not determined in the BLASTn analysis. In the tree, the eight isolates formed an exclusive clade that did not include sequences from any of the reference strains. This result provides further evidence that the eight isolates are the same species and phylogenetically distinct from all the reference strains (Figure 1).

**Figure 2:**
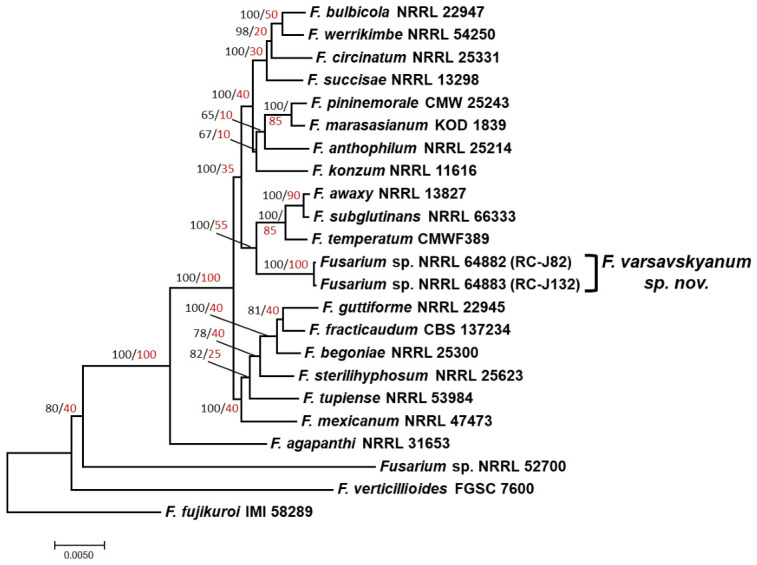
Species tree showing the relationships of strains NRRL 64882 (RC-J82) and NRRL 64883 (RC-J132), herein described as *Fusarium varsavskyanum* sp. nov., to reference strains of other members of the *Fusarium fujikuroi* species complex. The tree was inferred by maximum likelihood analysis of concatenated alignments of coding regions of 20 housekeeping genes. Numbers near branches are bootstrap values based on 1000 replicates (black type), or gene concordance values expressed as number of individual gene trees (out of 20) that included the branch (red type). The housekeeping genes and a summary of phylogenetic information derived from them are listed in Supplementary Table S2. A nexus formatted alignment file of the data is provided in Supplementary File S2.

To further assess whether the unidentified isolates are a phylogenetically distinct species of FFSC, we inferred a species tree from concatenated alginments of 20 full-length housekeeping genes retrieved from genome sequences of two of the isolates (NRRL 64882 and NRRL 64883) and reference strains of 21 other species of FFSC, including 18 species from the American clade. In the resulting species tree, NRRL 64882 and NRRL 64883 formed an exclusive and well-supported clade that was sister to a clade consisting of *F. awaxy*, *F. subglutinans*, and *F. temperatum* (Figure 2). Thus, trees inferred from the partial *TEF1* sequence, the combined dataset of *TEF1*, *RPB2*, *TUB2* and *CDM1* (Figure S1), and the 20 full-length housekeeping genes were consistent with NRRL 64882 and NRRL 64883 being strains of a novel species, hereinafter described as *Fusarium varsavskyanum* sp. nov., within the American clade of FFSC.

### 3.2. Distribution of Fusarium Species on Grasses

All *F. verticillioides* and *F. anthophilum* isolates, two *F. subglutinans* isolates, and most *F. proliferatum* isolates were recovered from grasses collected during the winter when inflorescences were not present. Because identification of these grasses to genus and species required inflorescences, we identified the host plants of these isolates only to the taxonomic level of the family Poaceae (Table 1). The remaining *F. proliferatum* isolates were recovered from *Leersia luziola*, *Paspalum notatum*, *Eragrostis* sp., *Hymenachme* sp. or *Diplacha* sp. Each of the four *F. pseudocircinatum* isolates was recovered from a different grass genus: *Spartina* sp., *Eriochloa* sp., *Chloris* sp., and *Cynodon dactilon*. One *F. subglutinans* isolate was obtained from *Elionurus* sp., while two others and the one *F. temperatum* isolate were recovered from *Panicum* sp. (Table 1).

### 3.3. Secondary Metabolite Biosynthetic Genes

BLAST analysis using sequences of known secondary metabolite biosynthetic genes to query genome sequences of *F. varsavskyanum* strains NRRL 64882 and NRRL 64883 revealed the distribution (i.e., presence and absence) of multiple biosynthetic genes or gene clusters required for production of mycotoxins, plant hormones, or other secondary metabolites that are produced by other *Fusarium* species. The distribution was identical in both *F. varsavskyanum* strains (Table 3). With respect to mycotoxins, the fusaric acid and beauvericin/enniatin biosynthetic gene clusters were detected in the two *F. varsavskyanum* genome sequences, but the fumonisin, fusarin, trichothecene, and zearalenone clusters were not detected. Biosynthetic gene clusters for the plant hormones auxins, cytokinins, and gibberellins were also detected in the *F. varsavskyanum* genome sequences. Additional gene clusters or individual genes that were detected were those that confer production of the polyketide-derived metabolites fusarubin and bikaverin, the nonribosomal peptides ferricrocin and fusarinine, and the terpenes eremophilene, koraiol, carotenoids, guai-6-10(14)-diene, and α-acorenol (Table 3). With three exceptions, the distribution of the genes/gene clusters in the *F. varsavskyanum* strains was the same as the distribution in the closely related species *F. subglutinans* and *F. temperatum* (Table 3). The exceptions were: (1) the six-gene depudecin cluster—*F. subglutinans* and *F. temperatum* had intact orthologs of the cluster, but both *F. varsavskyanum* strains had only a partial cluster that lacked the polyketide synthase gene (*DEP1*), which is critical for depudecin production; (2) the two-gene beauvericin/enniatin biosynthetic gene cluster—the *F. varsavskyanum* strains and *F. temperatum* had intact orthologs of this cluster, but as previously reported [54], the *F. subglutinans* ortholog of the nonribosomal peptide synthase gene (*NRPS22*) had multiple mutations that most likely rendered the gene nonfunctional; and (3) the six-gene fujikurin cluster—*F. temperatum* had an intact ortholog of this cluster, but the two *F. varsavskyanum* strains and *F. subglutinans* had none of the cluster genes.

### 3.4. Mycotoxin and Pigment Analysis

Extracts of maize kernel cultures of *F. varsavskyanum* strains NRRL 64882 and NRRL 64883 were subjected to a targeted HPLC-MS analysis of six secondary metabolites for which the corresponding biosynthetic gene clusters were identified in the genome sequences of the two strains. The six metabolites comprised three mycotoxins—beauvericin, enniatins, and fusaric acid—and three pigments—bikaverin, bostrycoidin, and fusarubin (Table 3). Despite the presence of the biosynthetic gene clusters in the strains, the only known secondary metabolite detected in culture extracts was fusaric acid. This mycotoxin was detected in extracts of the three replicate cultures of both strains at an average level of 1700 μg/g cracked maize kernel (range 1390–2190 μg/g). We also examined the culture extracts for the presence of moniliformin, a metabolite for which the biosynthetic genes have not been identified. Moniliformin was not detected in the culture extracts of either *F. varsavskyanum* strain.

### 3.5. Mating Type Identification and Sexual Stage Induction

Mating type idiomorphs (MAT-1/MAT-2) were identified for all *F. varsavskyanum* strains based on the results of a standard PCR assay. One strain (RC-J82 = NRRL 64882) carried the MAT-1 idiomorph, and the other seven carried the MAT-2 idiomorph. Analysis of genome sequences revealed that NRRL 64882 had the MAT-1 idiomorph with the *MAT1-1-1* and *MAT1-1-2* genes, and strain NRRL 64883 had the MAT-2 idiomorph with the *MAT1-2-1* and *MAT1-2-3* genes. None of the attempted mating crosses between isolates of *F. varsavskyanum* or between isolates of *F. varsavskyanum* and mating type tester strains of *F. subglutinans* resulted in the formation of perithecia after 10 weeks of incubation.

### 3.6. Taxonomy

**Figure 3:**
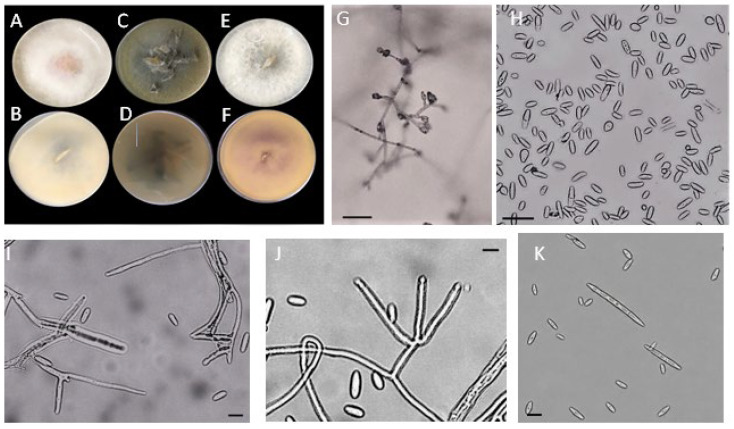
Macroscopic characteristics of *Fusarium varsavskyanum* NRRL 64882. (**A**,**B**). Colony on Spezieller Nährstoffarmer agar. (**A**). Colony surface. (**B**). Colony reverse. (**C**,**D**). Colony on carnation leaf agar. (**C**). Colony surface. (**D**). Colony reverse. (**E**,**F**). Colony color on potato dextrose agar. (**E**). Colony surface. (**F**). Colony reverse. (**G**). Microconidia produced on mono- and polyphialides and aggregated in false heads and palisade. (**H**). Microconidia, usually unicellular, ovoid 0 to 1 septate, mostly 0 septate, and piriform. (**I**). Polyphialides (**J**). Branched monophialides. (**K**). Mesoconidia. Bars: (**G**,**H**) = 50 μm; (**I**–**K**) = 10 μm.

***Fusarium varsavskyanum*** E. Cendoya, M. J. Nichea, C. Romero, R.H. Proctor & M.L Ramirez sp. nov. (Figure 3).

MycoBank: MB 846905

Types: ARGENTINA, CHACO PROVINCE Chaco Wetlands, Ramsar site no. 1366 (S 27°30′56.8″ W 59°05′22.9″), originally isolated from Poaceae plants, July 2011, Maria L. Ramirez RC-J132 (holotype RCVC 9963, a dry culture of RC-J132, NRRL 64883 Herbarium of the Natural Sciences Department, National University of Río Cuarto, Córdoba, Argentina). Ex-type culture NRRL 64883 = RC-J132. GenBank: *TEF1* = OQ134082; *RPB2* = OQ134078; *TUB2* = OQ134093; *CDM1* = OQ134088. COMPLETE GENOME = JBNHUV000000000.

Diagnosis*: F.** varsavsk**yanum* resembles *F. subglutinans*. Colony, false head morphology, polyphialides and rate of growth can overlap and so are not reliable criteria. Thus, the presence of abundant microconidia with piriform shape is the main marker to use to distinguish *F. varsavskyanum* from *F. subglutinans*.

Etymology: Named in honor of the late Dr. Edith Varsavsky, pioneer in the study of mycotoxicogenic fungi and mycotoxins in Argentina.

Description: Colonies on PDA under 12/12 h photoperiod cold white and black-fluorescent lamps at 25 °C growing rapidly, reaching 3.4 cm at 25 °C in 4 days, pink or vinaceous to violet; aerial mycelium abundant (Figure 3).

Microscopic characters: On CLA and SNA sporodochia can be present, when present they are tan to orange. Aerial mycelium floccose. Conidiophores usually erect and branched. Macroconidia abundant, falcate to rather straight, 3-5-septate, with a distinct foot-cell, (54.1–)54.3–67.4(–85.5) × (5.3–)5.4–6.3(–7.4) µm in total range, 65.3 ± 14.8 × 6.6 ± 1.0 on average ± SD (*n* = 20). Mesoconidia are present: (29.9–)31.5–41.5(–44.9) × (5.7–)5.9–8.1(–8.3) µm in total range, 36.3 ± 4.4 × 6.7 ± 0.9 on average ± SD (*n* = 21). Microconidia produced on mono- and polyphialides and aggregated in false heads and palisade, usually unicellular, ovoid 0 to 1 septate, mostly 0 septate: (10.5–)11.2–19.5(–20.2) × (5.0–)5.1–7.5(–7.7) µm in total range, 15.1 ± 2.3 × 6.4 ± 0.6 on average ± SD (*n* = 51)., and piriform: (10.2–)10.6–18.6(–18.7) × (7.1–)7.9–11.5(–11.7) µm in total range, 14.4 ± 2.1 × 9.6 ± 1.1 on average ± SD (*n* = 58). Chlamydospores absent.

Distribution: Argentina

Additional strains examined: RC-J251, RC-J224, RC-J1448 and RC-J82 = NRRL 64882.

Most *F. varsavskyanum* isolates were isolated from grasses collected during winter, when inflorescences were not present. As a result, identification of the grasses to genus and species was not possible. For one isolate, however, the identity of the grass host was determined to be *Elionorus* sp. (Table 1).

## 4. Discussion

The results of the current study indicate that isolates of FFSC recovered from asymptomatic wild grasses collected from the Chaco Wetlands of Argentina included both previously described species and one novel species, *F. varsavskyanum.* As far as we are aware, our results are the first report that wild grasses in Argentina can harbor previously described FFSC species that cause disease and mycotoxin contamination of agricultural crops. The mycotoxin-producing pathogenic species were *F. proliferatum*, *F. pseudocircinatum*, *F. subglutinans*, *F. temperatum* and *F. verticillioides*. The occurrence of these species as endophytes in wild grasses from the Chaco Wetlands is consistent with a previous study in which multiple mycotoxins produced by members of the FFSC were detected in the same grass samples from which the *Fusarium* isolates were recovered [4].

Our results are also consistent with previous studies showing that asymptomatic grasses in other parts of the world can be infected with pathogenic *Fusarium* species, such as *F. circinatum* [21], *F. graminearum* and *F. verticillioides* [55]. Our results also add to information that natural ecosystems can harbor novel *Fusarium* species and are potential inoculum reservoirs of plant pathogenic and mycotoxigenic *Fusarium* species [25]. To our knowledge, *F. varsavskyanum* is the second novel species of *Fusarium* recovered from wild grasses in the Chaco Wetlands.

Phylogenetic analysis of partial sequence of *TEF1*, *RPB2*, *TUB2* and *CDM1* from eight isolates as well as 20 housekeeping genes retrieved from genome sequence data of NRRL 64882 and NRRL 64883 provided support for the identification of *F. varsavskyanum* as a novel species within the American clade of FFSC. The Americas are the geographic origin of *Elionorus* species, the only grass sample contaminated with *F. varsavskyanum* that could be identified to the level of genus. This is consistent with the phylogeographic hypothesis that divided FFSC species into three major clades (African, American and Asian) that corresponded to the geographic origin of each clade [10]. *Fusarium varsavskyanum* is phylogenetically distinct from previously described species in the American clade. It differs from *F. subglutinans* in morphology of microconidia; both species produce ovoid microconidia, but *F. varsavskyanum* also produces abundant piriform microconidia. This difference is a key morphological marker to distinguish *F. varsavskyanum* from *F. subglutinans*. Colony characters and macroconidia shape are not sufficiently different to distinguish the two species, and neither species produced chlamydospores.

Results of the MAT PCR assay revealed that each *F. varsavskyanum* strain contains a MAT-1 or MAT-2 idiomorph, which suggests that this fungus has the genetic potential for a heterothallic sexual reproductive mode. Therefore, the failure of *F. varsavskyanum* strains to cross on carrot agar might be because the experimental conditions employed were suboptimal for the novel species or the strains that we isolated were not female-fertile. Low percentages of female-fertile isolates have been reported in several species within the FFSC [56,57]. The failure of *F. varsavskyanum* isolates to cross as a male parent with a female fertile tester strain of *F. subglutinans* provides additional evidence for reproductive isolation of these two species.

The analysis of distribution of biosynthetic gene clusters in the genome sequences revealed that the gene cluster content of *F. varsavskyanum* was similar to two of its closest relatives, *F. subglutinans* and *F. temperatum* (Table 3). The analysis of gene clusters indicated that *F. varsavskyanum* has the genetic potential to produce the plant hormones auxins, cytokinins and gibberellins. Production and/or potential to produce these plant hormones has been reported previously in some other members of FFSC, including *F. fujikuroi* and *F. proliferatum*, and could contribute to endophytic growth by enhancing growth and development of the plant hosts [58,59]. The presence in the *F. varsavskyanum* genomes of the gene clusters that confer production of the pigments bikaverin, carotenoids and fusarubins was expected given that the bikaverin cluster occurs widely among members of FFSC, and the carotenoid and fusarubin clusters occur widely in the genus *Fusarium* [58,60,61,62]. These pigments have potential to provide protection from solar radiation, but bikaverin and fusarubin also exhibit anti-bacterial and anti-fungal activity [63]. The genome sequence analysis indicated that *F. varsavskyanum* has the genetic potential to produce only three mycotoxins, beauvericin, enniatins and fusaric acid, which are considered emerging mycotoxins because they are not regulated and their impact on health of humans, pets and livestock is poorly understood [64]. It is notable the *F. varsavskyanum* genome sequences lacked genes that confer production of the mycotoxins fumonisins, trichothecenes, and zearalenone, which are among the mycotoxins of most concern to food and feed safety. Based on these findings, *F. varsavskyanum* does not have potential to cause contamination of wild grasses or crops with mycotoxins of major concern. The presence of beauvericin/enniatin biosynthetic genes in the *F. varsavskyanum* genome sequences indicates that it is one of the potential contributors to the previously observed beauvericin contamination in grasses from the Chaco Wetland [4].

Although the genome of *F. varsavskyanum* included gene clusters that confer production of six secondary metabolites for which we had standards, the HPLC-MS analysis indicated that strains NRRL 64882 and NRRL 64883 produced only fusaric acid under the culture conditions used in the current study. The finding that *F. varsavskyanum* strains do not produce the metabolic products of biosynthetic gene clusters present in their genome is not surprising. Indeed, lack of production of secondary metabolite products of gene clusters in fungi is common under laboratory conditions [65]. Different substrates on which *Fusarium* strains are grown can affect whether or not the strains produce secondary metabolites [66]. Thus, *F. varsavskyanum* strains might produce secondary metabolites other than fusaric acid if they were grown on other substrates. A possible cause for lack of production of a metabolite even though the corresponding gene cluster is present is that the cluster genes are not expressed under some laboratory conditions, but natural habitat(s) of fungi include conditions that induce expression of the genes.

Although recovery of members of FFSC from asymptomatic grasses suggests that the fungi can exist as endophytes in the grasses, most of the previously described FFSC species that were recovered are plant pathogens of one or more agricultural crops. But previous studies also indicate that some members of the FFSC can exist as either endophytes or pathogens of crops depending on environmental conditions [67,68]. Thus, it is possible that the grass isolates examined in this study, or at least some of them, are pathogenic on crops and/or on wild grasses at some point during the grass lifecycles or under environmental conditions that did not exist when the grasses were sampled [4]. Because we did not assess the potential pathogenicity of the grass isolates in this study, it remains to be determined whether they are pathogenic on crops and/or wild grasses. At least four of the FFSC species recovered from the grasses are maize pathogens: *F. proliferatum*, *F. subglutinans*, *F. temperatum* and *F. verticillioides*. This is potentially significant given the importance of maize to agriculture in Argentina. Although no commercial maize fields are located in or near the Chaco wetlands, wild Poaceae plants located closer to agricultural fields have potential to serve as a source of inoculum for these pathogenic species. *F. proliferatum* and *F. verticillioides* are the two most important contributors to fumonisin contamination in maize [6]. *Fusarium subglutinans* is also a pathogen of maize, but it cannot produce FBs. The host range of *F. subglutinans* is not clear, but it includes teosinte and native North American grasses, as well as a range of other monocots and dicots. *Fusarium temperatum* was first described in 2011 as a species closely related to *F. subglutinans* and causes diseases in maize, including seedling blight, stalk rot, and ear rot [69]. *Fusarium temperatum* has also been isolated from sorghum [70], wheat [71] and recently from cruciferous plants [72]. This fungus produces beauvericin, moniliformin and fusaproliferin [73].

To our knowledge, this study is the first report of the occurrence of *F. pseudocircinatum* in Argentina. This species is broadly distributed across ecologically diverse habitats, and it is an important plant pathogen that infects several wild and cultivated plants [27]. *Fusarium pseudocircinatum* has been associated with mango malformation in Mexico and the Dominican Republic [74,75] and with stunting and malformation of sunflower plants in Brazil [76]. In Argentina, sunflowers are an important crop, whereas mango production occurs on a small scale. Isolates of *F. pseudocircinatum* are reported to produce moniliformin, fusaric acid, and beauvericin [77].

To our knowledge, this study is also the first report of the occurrence of *F. anthophilum* in Argentina. This species is also widely distributed and has been recovered from several plant species (orchids, millet, wheat and rice) in temperate regions of the world [27,78]. Although, *F. anthophilum* has not been associated with plant disease, recently it was reported to cause drying on South American jelly palm (*Butia odorata*) in Brazil [79]. In Australia, *F. anthophilum* has been recovered from the native grasses *Austrostripa aristiglumis* [80] and *Sorghum leiocladium* [81]. The fungus is not associated with any human or animal diseases. However, some strains have been reported to produce the mycotoxins moniliformin, fumonisins, beauvericin and fusaproliferin [5,17]. It is noteworthy that *F. anthophilum* predominantly produces the C series of fumonisins rather than the B series [6]. Therefore, although this species occurs in Chaco Wetland grasses and produces fumonisin, it is unlikely to contribute to the previously reported fumonisin B_1_ contamination in the grasses [4].

Because of their ability to produce fumonisins, *F. proliferatum* and *F. verticillioides* are likely causes of the previously reported fumonisin contamination in Chaco Wetland grasses [4]. In addition, *F. proliferatum*, *F. temperatum*, *F. anthophilum* and *F. pseudocircinatum* could be the main source of beauvericin in those grasses.

There is a growing body of evidence indicating that natural ecosystems can serve as reservoirs of inoculum of both previously recognized and novel pathogens of agricultural crops [21,26,81,82,83,84]. Burges [26] suggested that weeds and wild plants near and in agricultural fields could serve as alternative hosts for fungal pathogens of crops and, therefore, impact epidemics of crop diseases. In Australia, natural ecosystems are a rich source of genetic diversity of *Fusarium* species and a reservoir of known and potential pathogens. Burgess [26] stated that studies of *Fusarium* in these ecosystems can contribute to a better understanding of the origin and ecology of novel pathogens at the species or subspecies level. He emphasized that extensive systemic surveys of *Fusarium* species are needed in natural ecosystems of Africa, Asia and South America. Thus, the current and previous [7,8] studies on *Fusarium* species in Chaco Wetland grasses contribute to understanding the potential impact of natural ecosystems on agriculture in South America. Similarly, Mourelos et al. [83] found that *Fusarium graminearum*, the predominant cause of Fusarium head blight of wheat in Argentina, occurs frequently in gramineous and non-gramineous weeds near fields of cereal crops in the Argentinian province of Buenos Aires.

Together, our results add to data on potential causes of mycotoxin contamination and fungal communities in natural grassland ecosystems. Moreover, the recovery of *F. chaquense* [7] and *F. varsavskyanum* from Chaco Wetlands grasses suggests that this important natural ecosystem in Argentina could harbor additional novel microbial species.

## Figures and Tables

**Table 1:** Information on isolates of the *Fusarium fujikuroi* species complex recovered from Chaco Wetland grasses.

*Fusarium* Species	Strain	Month Year	Host	GPS Coordinates	GenBank Accession No.			
					*TEF1*	*RPB2*	*CMD1*	*TUB2*
*F. anthophilum*	RC-J249	July 2011	Poaceae	S 27°31′08.4″ W 59°04′37.6″	MT123767			
	RC-J140	July 2011	Poaceae	S 27°31′10.7″ W 59°04′36.3″	MT123777			
*F. proliferatum*	RC-J72	July 2011	Poaceae	S 27°31′09.2″ W 59°04′36.8″	PZ453710			
	RC-J268	July 2011	Poaceae	S 27°31′10.8″ W 59°04′34.9″	PZ453711			
	RC-J310	July 2011	Poaceae	S 27°30′58.1″ W 59°05′23.7″	PZ453712			
	RC-J311	July 2011	Poaceae	S 27°30′58.1″ W 59°05′23.7″	PZ453713			
	RC-J397	July 2011	Poaceae	S 27°31′41.7″ W 59°04′33.1″	PZ453714			
	RC-J406	July 2011	Poaceae	S 27°31′41.8″ W 59°04′32.4″	PZ453715			
	RC-J412	July 2011	Poaceae	S 27°31′41.7″ W 59°04′31.6″	PZ453716			
	RC-J437	July 2011	Poaceae	S 27°31′41.7″ W 59°04′32.3″	PZ453717			
	RC-J459	July 2011	Poaceae	S 27°31′26.2″ W 59°05′00.4″	PZ453718			
	RC-J484	July 2011	Poaceae	S 27°31′25.8″ W 59°04′59.9″	PZ453719			
	RC-J506	July 2011	Poaceae	S 27°34′16.4″ W 60°23′42.4″	PZ453720			
	RC-J701	July 2011	Poaceae	S 27°33′34.5″ W 60°25′02.1″	PZ453721			
	RC-J739	July 2011	Poaceae	S 27°33′52.7″ W 60°24′39.6″	PZ453722			
	RC-J761	July 2011	Poaceae	S 27°34′53.1″ W 60°24′43.0″	PZ453723			
	RC-J404	July 2011	Poaceae	S 27°31′41.7″ W 59°04′31.6″	PZ453724			
	RC-J1689	February 2014	Poaceae	S 27°31′04.1″ W 59°05′05.6″	PZ453725			
	RC-J630	July 2011	Poaceae	S 27°33′28.3″ W 60°25′01.5″	PZ453726			
	RC-J781	July 2011	Poaceae	S 27°33′28.8″ W 60°25′00.3″	PZ453727			
	RC-J267	July 2011	Poaceae	S 27°31′10.8″ W 59°04′34.9″	PZ453728			
	RC-J1718	September 2014	*Leersia luziola*	S 27°31′06,9″ W 59°05′01.0″	MT123773			
	RC-J1782	September 2014	*Paspalum notatum*	S 27°30′39.0″ W 59°04′58.7″	PZ453729			
	RC-J443	July 2011	Poaceae	S 27°31′41.2″ W 59°04′32.7″	PZ453730			
	RC-J421	July 2011	Poaceae	S 27°31′42.9″ W 59°04′31.5″	PZ453731			
	RC-J425	July 2011	Poaceae	S 27°31′42.9″ W 59°04′31.5″	PZ453732			
	RC-J454	July 2011	Poaceae	S 27°31′26.8″ W 59°05′00.4″	PZ453733			
	RC-J466	July 2011	Poaceae	S 27°31′25.6″ W 59°05′00.5″	PZ453734			
	RC-J622	July 2011	Poaceae	S 27°33′28.2″ W 60°25′05.3″	PZ453735			
	RC-J635	July 2011	Poaceae	S 27°33′28.8″ W 60°25′00.3″	PZ453736			
	RC-J733	July 2011	Poaceae	S 27°33′51.7″ W 60°24′41.0″	PZ453737			
	RC-J1665	September 2014	*Diplacha* sp.	S 27°31′07.0″ W 59°05′00.9″	MT123775			
	RC-J1763	September 2014	*Hymenachme* sp.	S 27°30′39.0″ W 59°04′58.7″	PZ453738			
	RC-J1787	September 2014	*Dichantium* sp.	S 27°30′34.5″ W 59°05′02.7″	PZ453739			
	RC-J1789	September 2014	*Dichantium* sp.	S 27°30′34.5″ W 59°05′02.7″	PZ453740			
*F. pseudocircinatum*	RC-J1560	September 2014	*Shcloris* sp.	S 27°30′49.5″ W 59°05′06.1″	MT123782			
	RC-J1592	September 2014	*Spartina* sp.	S 27°30′49.5″ W 59°05′06.1″	MT123774			
	RC-J1519	September 2014	*Eriochloa* sp.	S 27°30′49.5″ W 59°05′06.1″	MT123771			
	RC-J1639	September 2014	*Cynodon dactylon*	S 27°30′49.5″ W 59°05′06.1″	MT123783			
*F. subglutinans*	RC-J401	July 2011	Poaceae	S 27°31′ 41.7″ W 59°04′33.1″	MT123768			
	RC-J1483	September 2014	*Elionurus* sp.	S 27°30′49.7″ W 59°05′06.3″	MT123770			
	RC-J1386	February 2014	*Panicum* sp.	S 27°30′50.3″ W 59°05′06.4″	MT123780			
	RC-J1387	February 2014	*Panicum* sp.	S 27°30′50.3″ W 59°05′06.4″	MT123781			
*F. temperatum*	RC-J1609	September 2014	*Panicum* sp.	S 27°30′50.3″ W 59°05′06.4″	MT123772			
*F. varsavskyanum* sp. nov.	RC-J34	July 2011	Poaceae	S 27°34′ 16.4″ W 60°23′42.4″	PZ453703			
	RC-J82, NRRL 64882	July 2011	Poaceae	S 27°34′ 14.0″ W 60°23′47.4″	OQ134079	OQ134074	OQ134084	OQ134089
	RC-J217	July 2011	Poaceae	S 27°34′ 02.3″ W 60°23′45.6″	PZ453704			
	RC-J224	July 2011	Poaceae	S 27°34′ 02.3″ W 60°23′45.6″	OQ134080	OQ134075	OQ134085	OQ134090
	RC-J251	July 2011	Poaceae	S 27°34′ 01.8″ W 60°23′43.2	OQ134081	OQ134076	OQ134086	OQ134091
	RC-J132^T^, NRRL 64883	July 2011	Poaceae	S 27°34′ 05.7″ W 60°23′48.6″	OQ134082	OQ134078	OQ134088	OQ134093
	RC-J1448	February 2014	*Elionurus* sp.	S 27°30′49.7″ W 59°05′06.3″	OQ134083	OQ134077	OQ134087	OQ134092
	RC-J279	July 2011	Poaceae	S 27°31′ 08.2″ W 59°04′38.2″	PZ453707			
*F. verticillioides*	RC-J424	July 2011	Poaceae	S 27°31′42.9″ W 59°04′31.5″	MT123769			
	RC-J22	July 2011	Poaceae	S 27°31′10.6″ W 59°04′38.4″	MT123776			
	RC-J413	July 2011	Poaceae	S 27°31′41.7″ W 59°04′31.6″	MT123778			
	RC-J490	July 2011	Poaceae	S 27°31′25.9″ W 59°05′00.5″	MT123779			
	RC-J657	July 2011	Poaceae	S 27°33′ 34.2″ W 60°24′59.7″	PZ453708			
	RC-J745	July 2011	Poaceae	S 27°33′ 53.5″ W 60°24′36.2″	PZ453709			

**Table 2:** Source information for *TEF1* reference sequences used in phylogenetic analyses.

*Fusarium* Species	Strain Number	Host	Origin	GenBank Accession Number *TEF1*
*F. agapanthi*	NRRL 54464	*Agapanthus* sp.	Australia	MN193856
*F. anthophilum*	NRRL 13602	*Hippeastrum* sp.	Germany	AF160292
*F. awaxy*	CBS 139380	*Corn stalk*	USA	MN534058
*F. begoniae*	NRRL 25300	*Begonia elatior*	Germany	AF160293
*F. bulbicola*	NRRL 13618	*Nerine bowdenii*	Germany	AF160294
*F. circinatum*	NRRL 25331	*Pinus radiata*	USA	AF160295
*F. fracticaudum*	CMW 25245	*Pinus maximinoii*	Colombia	KJ541059
*F. fujikuroi*	NRRL 13566	*Oryza sativa*	Taiwan	AF160279
*F. guttiforme*	NRRL 22945	*Ananas comosus*	England	AF160297
*F. konzum*	MRC 8544	*Sorghastrum nuttans*	USA	EU220235
*F. marasasianum*	CMW 25261	*Pinus patula*	Colombia	KJ541063
*F. mexicanum*	NRRL 47473	*Mangifera indica inflorescence*	Mexico	GU737416
*F. oxysporum*	NRRL 22902	*Pseudotsuga menziesii*	USA	AF160312
*F. pininemorale*	CMW 25243	*Pinus tecunumanii*	Colombia	KJ541064
*F. proliferatum*	NRRL 66424	Tropical rain forest soil	Papua New Guinea	MN534059
*F. pseudocircinatum*	NRRL 22946	*Solanum* sp.	Ghana	AF160271
*F. subglutinans*	NRRL 22016	*Zea mays*	USA	HM057336.1
*F. sterilihyphosum*	NRRL 25623	Mango	South Africa	AF160300
*F. succisae*	NRRL 13613	*Succisa pratensis*	Germany	AF160291
*F. temperatum*	NRRL 25622	*Zea mays*	South Africa	AF160301
*F. tupiense*	NRRL 53984	*Mangifera indica*	Brazil	GU737404
*F. verticillioides*	NRRL 22172	*Zea mays*	Germany	AF160262
*F. werrikimbe*	F19350 = CBS 125535	*Sorghum leiocladum*	Australia	EF107131

NRRL: ARS Culture Collection, United States Department of Agriculture; CBS: Culture Collection of the Westerdijk Fungal Biodiversity Institute, Utrecht, the Netherlands; CMW: Culture Collection of the Forestry and Agricultural Biotechnology Institute (FABI), University of Pretoria, Pretoria, South Africa; MRC: Medical Research Council, Tygerberg, South Africa.

**Table 3:** Distribution of secondary metabolite biosynthetic genes/gene clusters in *F. varsavskyanum* sp. nov. strains NRRL 64882 and NRRL 64883 and one representative strain each of the related species *F. subglutinans* and *F. temperatum*.

	*F. varsavskyanum* sp. nov.		*F. subglutinans*	*F. temperatum*
Metabolite Gene/Gene Cluster ^a^	NRRL 64882	NRRL 64883	NRRL 66333	CFWF389
**Polyketides**				
2-AOD-3-o	No	No	No	No
Aurofusarin	No	No	No	No
Bikaverin	Yes	Yes	Yes	Yes
Depudecin	Partial	Partial	Yes	Yes
Equisetin	Partial	Partial	Partial	Partial
Fujikurin	No	No	No	Yes
Fumonisin	No	No	No	No
Fusaric Acid	Yes	Yes	Yes	Yes
Fusaridione	No	No	No	No
Fusarielin	No	No	No	No
Fusarin	No	No	No	No
Fusarubin	Yes	Yes	Yes	Yes
Gibepyrone	Yes	Yes	Yes	Yes
Zearalenone	No	No	No	No
**Non-ribosomal peptides**				
Apicidin	No	No	No	No
Beauvericin/Enniatin	Yes	Yes	No	Yes
Ferricrocin	Yes	Yes	Yes	Yes
Fusarinine	Yes	Yes	Yes	Yes
Gramilin	No	No	No	No
Malonichrome	No	No	No	No
**Polyketide-Non-ribosomal peptides**				
Fusaristatin	No	No	No	No
W493-B	No	No	No	No
**Terpenes**				
a-Acorenol	Yes	Yes	Yes	Yes
Eremophilene	Yes	Yes	Yes	Yes
Guai-6,10(14)-diene	Yes	Yes	Yes	Yes
Koraiol	Yes	Yes	Yes	Yes
Carotenoids	Yes	Yes	Yes	Yes
Culmorin	No	No	No	No
Trichothecene	No	No	No	No
**Pant Hormones**				
Auxin	Yes	Yes	Yes	No
Cytokinin_Cluster 1	Yes	Yes	Yes	Yes
Cytokinin_Cluster 2	Yes	Yes	Yes	Yes
Gibberellin (=terpene)	Yes	Yes	Yes	Yes
**Other metabolites**				
Butenolide	No	No	No	No

^a^ For each metabolite, “No” indicates the biosynthetic gene or gene cluster was not detected; “Yes” indicates detected; and “Partial” indicates part of the cluster was detected. The genome sequences of *F. subglutinans* and *F. temperatum* used in this analysis were previously reported and had GenBank/NCBI accessions JAAOAV01 and LJGR01, respectively.

## Data Availability

All sequences generated in this study were deposited in GenBank, with the accession numbers provided in Table 1 and Table S1. The original contributions presented in this study are included in the article and Supplementary Materials. Further inquiries can be directed to the corresponding author.

## Supplementary Materials

The following supporting information can be downloaded at: https://www.mdpi.com/article/10.3390/jof12060444/s1, Figure S1: Bayesian phylogenetic tree inferred from the combined dataset *TEF1*, *RPB2*, *TUB2* and *CDM1* sequences showing NRRL 64882 and NRRL 64883 being members of the American clade of the FFSC and a novel species. Bayesian posterior probability scores ≥ 0.8 are shown at the internodes. *F. oxysporum* NRRL 22902 was used as the outgroup.; Table S1: Source information for *TEF1*, *RPB2*, *CMD1* and *TUB* reference sequences used in the phylogenetic analyses; Table S2: Summary of phylogenetic data for the 20 housekeeping genes used to infer the species phylogeny in this study. File S1: Fasta_formatted sequences of *Fusarium* secondary metabolite biosynthetic genes. File S2: Nexus-formatted file with concatenated and partitioned alignment of exon and intron sequences of 20 housekeeping genes from *F. varsavskyanum* strains RC-J82 (NRRL 64882) and RC-J132 (NRRL 64883) as well as reference strains of 21 other members of the *Fusarium fujikuroi* species complex.
