# Supplementary material for: Members of the Fusarium fujikuroi Species Complex Isolated from Asymptomatic Wetland Grasses in Argentina Include Previously Described Species Pathogenic on Cereal Crops and a Novel Species
Source: J Fungi (Basel). 2026 Jun 17;12(6):444. doi: 10.3390/jof12060444 (PMC13300770; doi:10.3390/jof12060444)
Supplement: Supplementary file 1 [file jof-12-00444-s001.zip › Supplementary Table S1.pdf]

Supplementary Table S1. Source information for *TEF1, RPB2, CMD1 and TUB* reference sequences used in the phylogenetic analyses

Fusarium Species	Strain	GenBank accession numbers			
		TEF1	*RPB2*	*CMD1*	*TUB*
F. agapanthi	NRRL 54463	KU900630	KU900625	KU900611	KU900635
F. anthophilum	CBS 222.76	MW402114	MW402811	MW402451	MW402312
F. awaxy	CBS 139380	MN534058	MN534238.1	MN534172.1	MN534107.1
F. begoniae	NRRL 25300	AF160293	MN193886.1	MT010899.1	U61621.1
F. bulbicola	NRRL 13618	AF160294	KF466404.1	AF158347.1	U61624.1
F. circinatum	NRRL 25331	AF160295	JX171623.1	AF158348.1	U61547.1
F. fujikuroi	NRRL 13566	AF160279	JX171570.1	AF158332.1	U34415.1
F. guttiforme	NRRL 22945	AF160297	JX171618.1	AF158350.1	U34475.1
F. konzum	CBS 119849	LT996098	MW402733	LT996182	MN534095
F. marasasianum	CMW 25261	KJ541063	MN534249.1	MN534208.1	KJ541054.1
F. mexicanum	NRRL 47473	GU737416	LR792615.1	GU737389.1	GU737362.1
F. oxysporum	NRRL 22902	AF160312	LT575065.1	AF158365.1	U34479.1
F. pininemorale	CMW 25243	KJ541064	MN534250.1	MN534211.1	MN534115.1
F. sterilihyphosum	NRRL 25623	AF160300	LR792617.1	AF158353.1	AF160344.1
F. subglutinans	NRRL 22016	HM057336.1	JX171599.1	AF158342.1	U34417.1
F. succisae	NRRL 13613	AF160291	MW402766	AF158344.1	U34419.1
F. temperatum	NRRL 25622	AF160301	LT970765	AF158354	AF160317
F. tupiense	NRRL 53984	GU737404	LR792619	GU737377	GU737296
F. varsavskyanum sp. nov.	RC-J82 T, NRRL 64882	OQ134079	OQ134074	OQ134084	OQ134089
F. varsavskyanum sp. nov.	RC-J224	OQ134080	OQ134075	OQ134085	OQ134090
F. varsavskyanum sp. nov.	RC-J251	OQ134081	OQ134076	OQ134086	OQ134091
F. varsavskyanum sp. nov.	RC-J132T, NRRL 64883	OQ134082	OQ134078	OQ134088	OQ134093
F. varsavskyanum sp. nov.	RC-J1448	OQ134083	OQ134077	OQ134087	OQ134092
