# Supplementary material for: Members of the Fusarium fujikuroi Species Complex Isolated from Asymptomatic Wetland Grasses in Argentina Include Previously Described Species Pathogenic on Cereal Crops and a Novel Species
Source: J Fungi (Basel). 2026 Jun 17;12(6):444. doi: 10.3390/jof12060444 (PMC13300770; doi:10.3390/jof12060444)
Supplement: Supplementary file 1 [file jof-12-00444-s001.zip › Supplementary Table S2.pdf]

**Supplementary Table S2.** Summary of phylogenetic data for the 20 housekeeping genes used to infer the species phylogeny in this study.

| Gene | Protein/Function | Locus_Tag[a] | GenBank Accession[a] | Length[b] | No. PICs[c] | % PICs[d] | % Total PICs[e] | Model[f] |
|---|---|---|---|---|---|---|---|---|
| *ACL1* | ATP citrate lyase large subunit | FVEG_04667 | XP_018749212 | 1470 | 44 | 3.0 | 1.7 | TN+F+G4 |
| *ACT1* | Actin | FVEG_00630 | XP_018742925 | 1419 | 106 | 7.5 | 4.1 | TNe+G4 |
| *CAL1* | Calmodulin | FVEG_07362 | XP_018753358 | 450 | 12 | 2.7 | 0.5 | TNe |
| *CPR1* | Cytochrome P450 Reductase | FVEG_06400 | XP_018751900 | 2079 | 62 | 3.0 | 2.4 | TNe+G4 |
| *DPA1* | DNA polymerase Alpha Subunit A | FVEG_16016 | XP_018752950 | 4380 | 179 | 4.1 | 6.9 | TNe+G4 |
| *DPE1* | DNA polymerase Epsilon Subunit A | FVEG_04699 | XP_018749259 | 6636 | 312 | 4.7 | 12.1 | TNe+G4 |
| *FAS1* | Fatty Acid Synthase Alpha Subunit | FVEG_04241 | XP_018748624 | 5568 | 232 | 4.2 | 9.0 | TN+F+G4 |
| *FAS2* | Fatty Acid Synthase Beta Subunit | FVEG_04242 | XP_018748627 | 6315 | 249 | 3.9 | 9.6 | TIM2+F+G4 |
| *KU70* | ATP-dependent DNA helicase 2 subunit 1 | FVEG_04235 | XP_018748618 | 1935 | 134 | 6.9 | 5.2 | TN+F+G4 |
| *LCB2* | Sphinganine Palmitoyl Transferase Subunit 2 | FVEG_10287 | XP_018757422 | 1992 | 86 | 4.3 | 3.3 | TN+F+I |
| *MCM7* | DNA Replication Licensing Factor | FVEG_08063 | XP_018754394 | 2445 | 94 | 3.8 | 3.6 | TNe+G4 |
| *PGK1* | Phoshpoglycerate Kinase | FVEG_10153 | XP_018757237 | 1257 | 58 | 4.6 | 2.2 | TN+F+I |
| *PHO5* | Phosphate Permease | FVEG_12069 | XP_018759883 | 1704 | 125 | 7.3 | 4.8 | TIM2+F+I+G4 |
| *RPB1* | RNA Polymerase Largest Subunit | FVEG_00683 | XP_018742997 | 5256 | 268 | 5.1 | 10.4 | TNe+I+G4 |
| *RPB2* | RNA Polymerase 2nd Largest Subunit | FVEG_09286 | XP_018756128 | 3813 | 210 | 5.5 | 8.1 | TIM3e+I+G4 |
| *TEF1* | Translation Elongation Factor 1alpha | FVEG_02381 | XP_018745816 | 1383 | 27 | 2.0 | 1.0 | TIM3+F+I |
| *TOP1* | Topoisomerase | FVEG_05113 | XP_018749972 | 2730 | 106 | 3.9 | 4.1 | TN+F+G4 |
| *TSR1* | Ribosomal Biogenesis Protein | FVEG_11260 | XP_018758717 | 2451 | 187 | 7.6 | 7.2 | TIM2e+I+G4 |
| *TUB1* | Tubulin alpha subunit | FVEG_00557 | XP_018742806 | 1350 | 50 | 3.7 | 1.9 | TN+F+I |
| *TUB2* | Tubulin beta subunit | FVEG_04081 | XP_018748359 | 1341 | 47 | 3.5 | 1.8 | TN+F+G4 |
| | | | | Σ = 55974 | Σ = 2588 | | Σ = 100 | |

[a] Locus-Tag and GenBank accession number for *F. verticillioides* ortholog of gene.
[b] Length, in nucleotides, of coding region in *F. verticillioides*.
[c] Number of parsimony-informative characters in coding region sequence.
[d] Percent of nucleotides in coding region sequence that are parsimony informative.
[e] Percent of total (2588) parsimony-informative characters in concatenated alignment.
[f] Best nucleotide substitution model for gene as determined with Bayesian Information Criterion as implemented in IQ-Tree version 1.6.12.
