# Supplementary figures and images for: Members of the Fusarium fujikuroi Species Complex Isolated from Asymptomatic Wetland Grasses in Argentina Include Previously Described Species Pathogenic on Cereal Crops and a Novel Species
Source: J Fungi (Basel). 2026 Jun 17;12(6):444. doi: 10.3390/jof12060444 (PMC13300770; doi:10.3390/jof12060444)

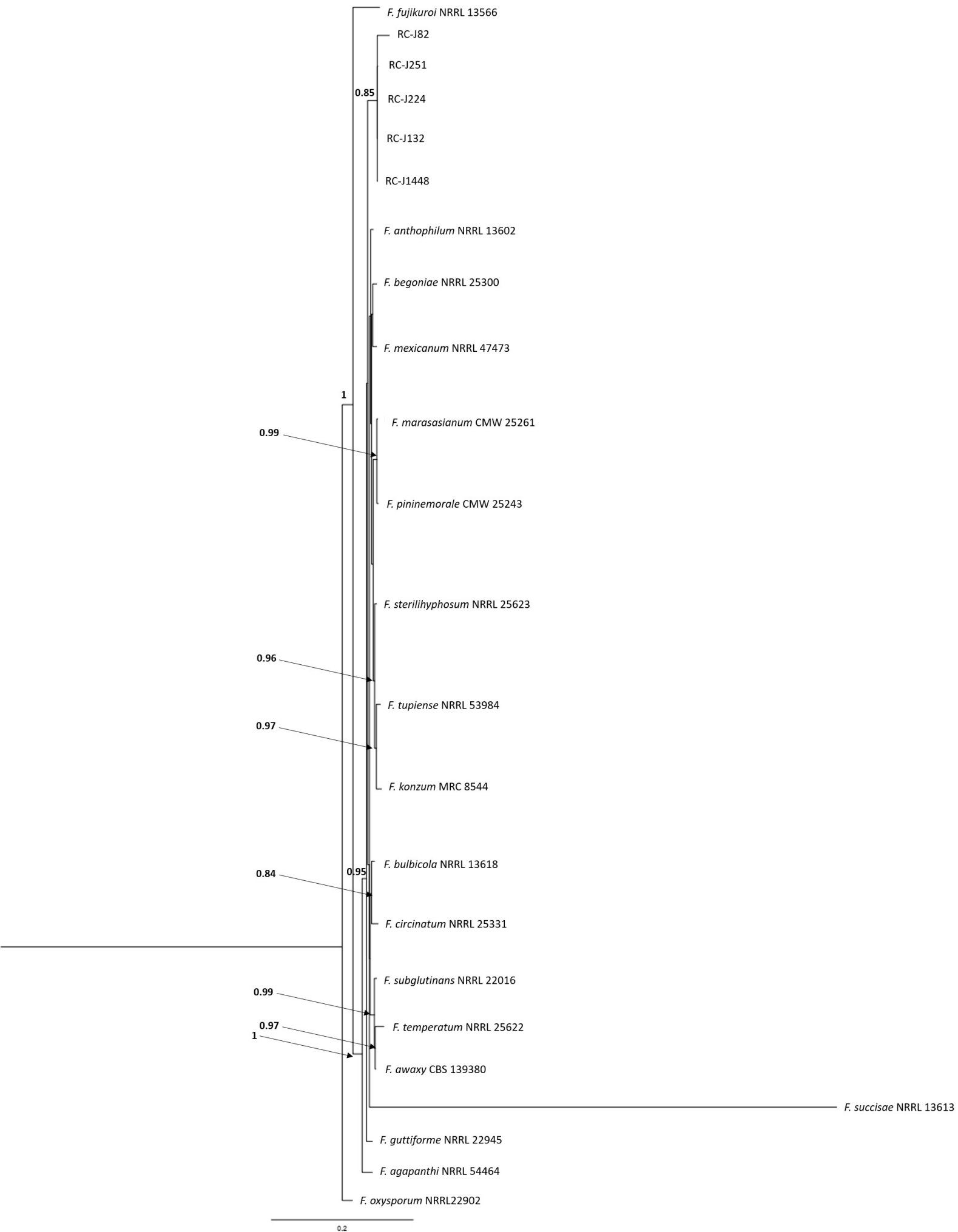

Supplement: Supplementary file 1 [file jof-12-00444-s001.zip › Supplementary Figure S1.pdf]
